# Current knowledge on non-steroidal anti-inflammatory drug-induced small-bowel damage: a comprehensive review

**DOI:** 10.1007/s00535-019-01657-8

**Published:** 2019-12-21

**Authors:** Toshio Watanabe, Yasuhiro Fujiwara, Francis K. L. Chan

**Affiliations:** 1 Department of Gastroenterology, Osaka City University Graduate School of Medicine, 1-4-3 Asahimachi, Abeno-ku, Osaka, 545-8585 Japan; 2 Department of Medicine and Therapeutics, Institute of Digestive Disease, The Chinese University of Hong Kong, Hong Kong SAR, People’s Republic of China

**Keywords:** Non-steroidal anti-inflammatory drug, Low-dose aspirin, Enteropathy, Innate immunity, Misoprostol

## Abstract

Recent advances in small-bowel endoscopy such as capsule endoscopy have shown that non-steroidal anti-inflammatory drugs (NSAIDs) frequently damage the small intestine, with the prevalence rate of mucosal breaks of around 50% in chronic users. A significant proportion of patients with NSAIDs-induced enteropathy are asymptomatic, but some patients develop symptomatic or complicated ulcers that need therapeutic intervention. Both inhibition of prostaglandins due to the inhibition of cyclooxygenases and mitochondrial dysfunction secondary to the topical effect of NSAIDs play a crucial role in the early process of injury. As a result, the intestinal barrier function is impaired, which allows enterobacteria to invade the mucosa. Gram-negative bacteria and endogenous molecules coordinate to trigger inflammatory cascades via Toll-like receptor 4 to induce excessive expression of cytokines such as tumor necrosis factor-α and to activate NLRP3 inflammasome, a multiprotein complex that processes pro-interleukin-1β into its mature form. Finally, neutrophils accumulate in the mucosa, resulting in intestinal ulceration. Currently, misoprostol is the only drug that has a proven beneficial effect on bleeding small intestinal ulcers induced by NSAIDs or low-dose aspirin, but its protection is insufficient. Therefore, the efficacy of the combination of misoprostol with other drugs, especially those targeting the innate immune system, should be assessed in the next step.

## Introduction

Non-steroidal anti-inflammatory drugs (NSAIDs) are widely prescribed for treatment of pain or inflammation in a variety of chronic conditions such as rheumatoid arthritis (RA) and osteoarthritis. NSAIDs exert these effects via the inhibition of cyclooxygenase (COX) and the resultant decrease in the synthesis of prostaglandins (PGs) [1]. One of the major adverse effects of NSAIDs is on the gastrointestinal (GI) tract. NSAIDs including low-dose aspirin (LDA), usually at dosages of 81–325 mg a day, can cause severe GI damage such as bleeding, perforation, and ulceration [2–4], which often limits the use of these drugs. Although it is known that NSAIDs have an injurious effect throughout the GI tract [5, 6], less attention had been paid to damage distal to the duodenum.

The introduction of new modalities such as capsule endoscopy [7] and balloon-assisted endoscopy [8] revealed that NSAIDs frequently injure the small bowel [9, 10], leading to great interest in the pathophysiology and treatment of NSAIDs-induced small intestinal damage. Although PG deficiency is a common key factor for NSAIDs-induced upper GI and small intestinal damages, there exist different pathophysiological mechanisms between these damages. Gastric acid plays a crucial role in NSAIDs-induced upper GI damage, whereas gut microbiome contributes to NSAIDs-induced enteropathy [11]. The latter implies that proton pump inhibitors (PPIs) are not effective against enteropathy, and therefore, distinct strategies for the NSAIDs-induced damages in these two regions are required. In this review, the epidemiology, pathophysiology, and treatment of NSAIDs-induced small intestinal damage are summarized, with a focus on recent data.

## Clinical features

### Endoscopic features

**Fig. 1:**
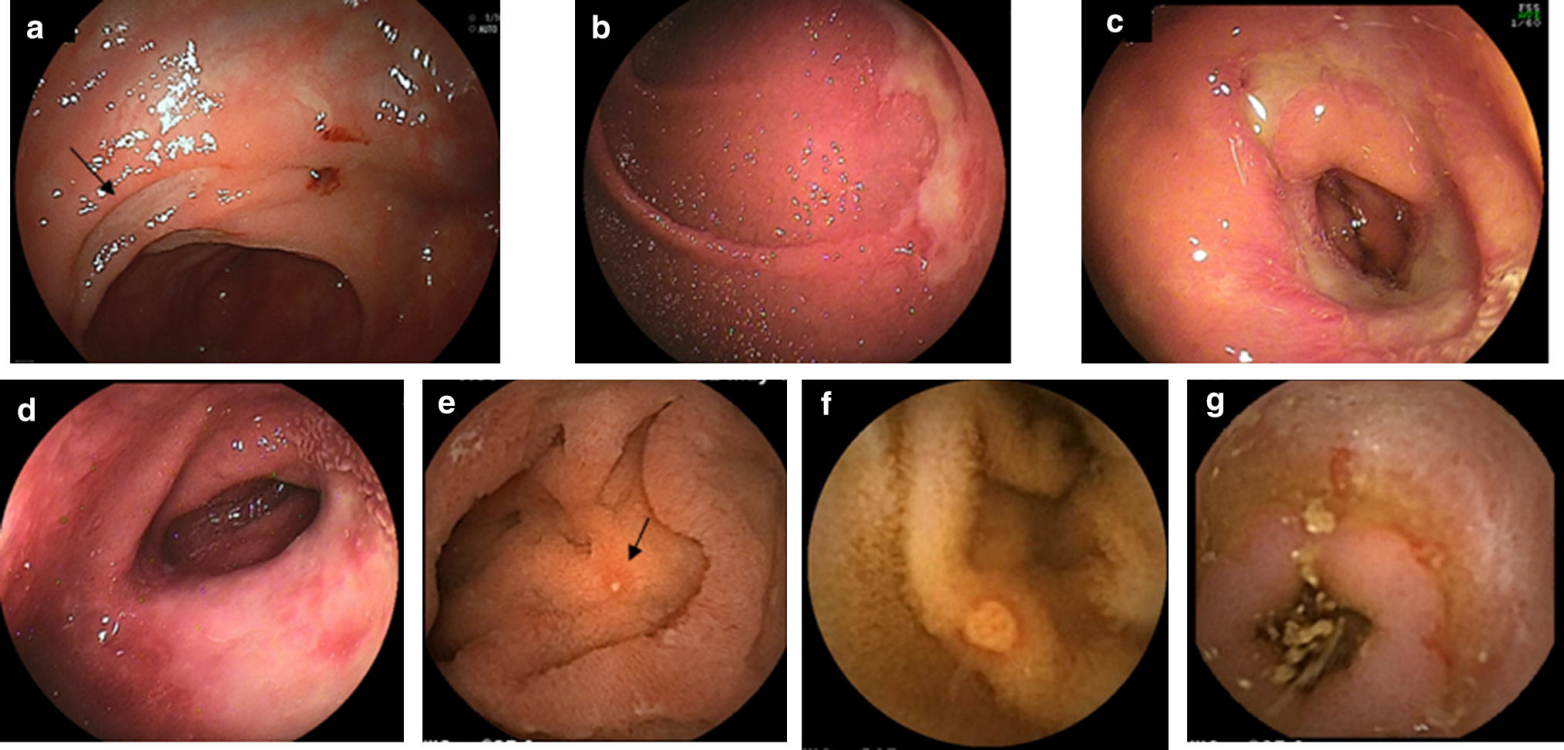
Endoscopic images of NSAIDs-induced small intestinal damage. **a**–**d** Images of balloon-assisted endoscopy. **a** oval-shaped (arrow), **b** longitudinal ulcer, **c** circular ulcer, and **d** diaphragm-like stricture. **e**–**g** Images of capsule endoscopy. **e** Erosion (arrow), **f** round ulcer, and **g** circular ulcer

Until the 21th century, diagnosis of NSAIDs-induced enteropathy was mainly made by indirect methods such as examining the fecal excretion of radio-labeled neutrophils and red blood cells [12], intestinal permeability test [13], and fecal calprotectin test [14]. The introduction of capsule endoscopy and balloon-assisted endoscopy at the beginning of this century enabled the direct visualization of the small bowel, and this has helped to clarify the characteristics of the NSAIDs-induced pathologies. NSAIDs induce various types of mucosal damage including red spots, erosions and round, oval-shaped, irregular, circular, and longitudinal ulcers and diaphragm-like stricture in the small intestine (Fig. 1) [10, 15]. In addition, multiple lesions occur commonly in the small bowel. Several studies conducted in western countries did not report any vulnerable sites of mucosal breaks (ulcers or erosions) in healthy volunteers who received short-term administration of NSAIDs [16-18]. However, a Japanese study reported a significantly higher incidence of denuded areas and ulcers in the proximal and distal regions of the small intestine, respectively [19]. Furthermore, mucosal breaks were frequently observed in jejunum than in ileum in RA patients on long-term NSAIDs therapy [10]. These results suggest that the duration of NSAIDs therapy and the medical history of the subject may affect the distribution of damage.

### Clinical symptoms

The damages described above can cause complications such as overt bleeding, perforation, stricture with symptoms of acute or subacute obstruction (i.e., chronic colicky abdominal pain, abdominal distension, and recurrent vomiting), hypoalbuminemia [20], and occult bleeding that may lead to the development of iron-deficiency anemia [21]. In contrast, uncomplicated NSAIDs-induced enteropathy rarely leads to clinical symptoms. In prospective studies using capsule endoscopy to evaluate the injurious effect of NSAIDs on the small bowel, most subjects were asymptomatic despite the high incidence of intestinal pathologies [22, 23]. However, some patients on NSAIDs therapy develop abdominal symptoms such as dyspepsia and nausea that do not respond to treatment with acid suppressants. Thus, it is possible that some, but not all, intestinal damages can cause such symptoms.

### Capsule endoscopic evaluation of the severity of NSAIDs-induced enteropathy

Capsule endoscopy can detect small lesions. However, many patients on NSAIDs with such lesions were asymptomatic and showed no abnormality in laboratory parameters. Therefore, the clinical significance of NSAIDs-induced small-bowel pathologies detected by capsule endoscopy is under discussion. Some investigators used the Lewis score, a capsule endoscopic grading system for small intestinal mucosal inflammation and damage [24], to evaluate the severity of damage caused by NSAID [25, 26]. According to the Lewis score, small-bowel inflammatory changes can be categorized into three groups: normal or clinically insignificant change, mild mucosal inflammatory change, and moderate or severe change. However, no studies reported the correlation of these groups with the clinical measures employed in patients with NSAIDs-induced enteropathy. Another classification system for NSAIDs-induced damage is based on categorizing small-bowel pathologies as normal, red spot, small erosion, large erosion, and ulcer. The endoscopic findings for these categories are scored from 0 to 4, where (0) indicates normal; (1), red spots; (2), 1–4 erosions; (3), > 4 erosions; and (4), large erosions/ulcers [9]. The initial scores were classified into three levels based on the severity of the damage—no damage (0–1), mild damage (2), and severe damage (3–4)—and the patients with severe damage had significantly lower hemoglobin concentrations and an insignificant trend for lower serum albumin levels compared to those with no damage [27]. Thus, severe damage, as defined by this classification, is a clinically significant condition that needs therapeutic intervention.

### Markers of the NSAIDs-induced small intestinal damage

Although capsule endoscopy is a noninvasive diagnostic technique that detects the presence and severity of the NSAIDs-induced small intestinal damages, it is expensive and time-consuming. Therefore, there is a need for simple noninvasive sensitive markers of NSAIDs-induced small intestinal damage. Urinary excretion of chromium-51-labeled ethylenediaminetetraacetic acid and fecal indium-111-labeled neutrophils were used to evaluate intestinal permeability and inflammation, respectively [6]. However, these radio-labeled methods are not used widely and a few or no comparative studies using these methods and capsule endoscopy have been conducted.

Calprotectin is a protein released by activated or damaged granulocytes, monocytes, and macrophages [28]. Since calprotectin is stable in feces, fecal calprotectin can be used as a biomarker of intestinal disorders, especially inflammatory diseases in the GI tract, such as inflammatory bowel diseases [29]. Several studies indicated the usefulness of fecal calprotectin as a biomarker of the NSAIDs-induced enteropathy. Maiden et al. reported that after 2 weeks of treatment with diclofenac, 27 of the 40 healthy volunteers (68%) had newly developed small-bowel pathologies, which were detected by capsule endoscopy and 75% of the subjects had elevated levels of fecal calprotectin [16]. However, their study failed to show either a significant correlation between the fecal calprotectin and capsule endoscopic results or a significant difference in the increase in the levels of fecal calprotectin between the subjects developing mucosal breaks and those that had no small-bowel abnormality. Furthermore, similar results were observed in a study wherein a 2-week treatment with NSAIDs (ibuprofen or celecoxib) in healthy volunteers significantly increased the fecal calprotectin levels, but no significant correlation was found between these levels and the number of small-bowel mucosal breaks [18]. These results suggest that the fecal calprotectin could not be used as a marker for the severity of NSAIDs-induced small intestinal damage or for monitoring the effect on the damage. Today, there are no biomarkers for the NSAIDs-induced enteropathy that can be used as an alternative to the direct evaluation by capsule endoscopy or balloon-assisted endoscopy.

## Epidemiology

### Prevalence of injury

In mid-2000s, a series of studies using capsule endoscopy reported the high potential of NSAIDs to cause injuries to the small intestine, regardless of the duration of the NSAID therapy. Graham et al. reported that small-bowel injury was seen in 71% of the patients with arthritis who took non-selective NSAIDs for more than 3 months, as compared to that of the 10% of controls (non-NSAIDs users) [9]. In another study with 28 RA patients, small intestinal mucosal breaks were detected in 13 of 16 patients (81%) who used NSAIDs for more than 12 months, whereas these mucosal breaks were detected only in 4 of 12 patients (33%) who did not use NSAIDs [10]. Prospective studies in the healthy volunteers who were given short-term non-selective NSAIDs treatment confirmed high toxicity of NSAIDs medication on the small bowel (Table 1) [16–18, 23, 30, 31].

**Table 1:** Capsule endoscopic prevalence of small-bowel mucosal breaks in subjects receiving NSAIDs or LDA

Author [Ref]	Year	N	Type of NSAIDs	Treatment period	Subject	Prevalence of mucosal breaks (%)
Graham et al. [9]	2005	21	Non-selective NSAIDs	> 3 months	Chronic user	62
Maiden et al. [16]	2005	40	Diclofenac (+ omeprazole)	2 weeks	Healthy volunteers	40
Goldstein et al. [17]	2005	111	Naproxen (+ omeprazole)	2 weeks	Healthy volunteers	55
		115	Celecoxib	2 weeks	Healthy volunteers	16
Goldstein et al. [18]	2007	112	Ibuprofen (+ omeprazole)	2 weeks	Healthy volunteers	26
		109	Celecoxib	2 weeks	Healthy volunteers	6
Maiden et al. [34]	2007	120	Non-selective NSAIDs	> 3 months	Chronic user	29
		40	Selective COX-2 inhibitors	> 3 months	Chronic user	22
Sugimori et al. [10]	2008	16	Non-selective NSAIDs	> 1 year	Chronic user	81
Hawkey et al. [30]	2008	45	Naproxen (+ omeprazole)	16 days	Healthy volunteers	78
		47	Lumiracoxib	16 days	Healthy volunteers	28
Fujimori et al. [23]	2009	15	Diclofenac (+ omeprazole)	2 weeks	Healthy volunteers	53
Maehata et al. [31]	2012	14	Celecoxib (+ omeprazole)	2 weeks	Healthy volunteers	43
		15	Meloxicam (+ omeprazole)	2 weeks	Healthy volunteers	27
Watanabe et al. [27]	2013	87	Non-selective NSAIDs	> 3 months	Chronic user	54
		21	Celecoxib	> 3 months	Chronic user	48
Watanabe et al. [37]	2008	11	Enteric-coated LDA (+ PPIs)	> 3 months	Chronic user with PUD	91
Sumecuol et al. [38]	2009	20	Enteric-coated LDA (+ esomeprazole)	2 weeks	Healthy volunteers	20
Endo et al. [39]	2009	22	LDA	> 3 months	Chronic user with OGIB	46*
Endo et al. [40]	2009	10	Enteric-coated LDA	2 weeks	Healthy volunteers	30
Hara et al. [41]	2018	45	LDA	> 2 weeks	Chronic user	51

*NSAID* non-steroidal anti-inflammatory drug, *LDA* low-dose aspirin, *COX* cyclooxygenase, *PPI* proton pump inhibitor, *PUD* peptic ulcer disease, *OGIB* obscure gastrointestinal bleeding

*Prevalence of small-bowel ulcer

The gastroduodenal safety profiles of selective COX-2 inhibitors were well established [32]. In recent years, the safety of these inhibitors on the small bowel has gained interest. At 2-week treatment with celecoxib, a selective COX-2 inhibitor caused fewer small intestinal injury than that with naproxen [17]. Similar results were reported by other studies [18, 31, 33], including a randomized, double-blinded trial that compared the small intestinal safety of lumiracoxib, another selective COX-2 inhibitor, to that of naproxen with a PPI in healthy volunteers [30]. Thus, selective COX-2 inhibitors are considered less injurious than non-selective NSAIDs for the small bowel, similar to the upper GI tract. However, Maiden et al. reported that the prevalence of small-bowel injuries including reddened folds, denuded areas, and mucosal breaks, was high in chronic users of the selective COX-2 inhibitors, and comparable to that in the chronic users of non-selective NSAIDs [34]. A cross-sectional study in RA patients also found no difference in the prevalence of mucosa breaks between long-term users of non-selective NSAIDs and celecoxib [27]. A large-scale, double-blinded, randomized, clinical trial over 6 months suggested that celecoxib is less likely to cause mucosal damage throughout the GI tract compared to diclofenac with a PPI [35]. However, the long-term use of selective COX-2 inhibitors may reduce its beneficial effects.

As described in detail below, the topical effect of NSAIDs on the small bowel is a key to induce intestinal damage [36]. Aspirin cannot exert the topical effect on the small bowel, because it is immediately absorbed in the stomach and duodenum, without entering the enterohepatic circulation. Together with the negative results in the clinical studies using intestinal permeability and fecal inflammatory markers [6], aspirin was believed to not cause any damage to the small bowel. Recently, Leung et al. reported a case of severe enteropathy induced by LDA, which led to a change in our perception toward the safety of aspirin on the lower GI tract and subsequent capsule endoscopic studies to assess the ulcerogenic potential of LDA. Surprisingly, capsule endoscopy identified mucosal breaks in 10 of 11 patients who took enteric-coated LDA for cardiovascular or cerebrovascular diseases with a maximum number of mucosal breaks being 33 [37]. Although prevalence rates of mucosal breaks varied depending on study design, the reported rates are as high as those in non-selective NSAIDs studies (Table 1) [38–41]. One reason for this very high incidence of injuries caused by enteric-coated LDA seems to be the amplified topical effect caused by exposure of the small-bowel mucosa to high concentrations of aspirin dissolved within the small bowel. In fact, the enteric-coated formulation of LDA was associated with higher prevalence of intestinal mucosal breaks, compared with that for buffered LDA [26].

### Risk factors for NSAIDs-induced enteropathy

In contrast to upper GI damage, risk factors for NSAIDs-induced small intestinal damage are not established. Recently, both laboratory and clinical studies demonstrated that PPI use may aggravate small intestinal injury caused by NSAIDs. Animal studies strongly suggest that enterobacteria, especially Gram-negative bacteria, are the most important factor for intestinal ulceration by NSAIDs [42]. Because gastric acid can kill bacteria in the stomach and the duodenum, acid suppression by PPIs affects the bacterial flora of the GI tract, thereby aggravating NSAIDs-induced enteropathy. Wallace et al. demonstrated that PPIs such as omeprazole and lansoprazole exacerbated NSAIDs-induced enteropathy by altering gut microbiota composition, which was characterized by the reduction of jejunal *Actinobacteria* and *Bifidobacteria* spp, in rats [43]. In a cross-sectional study, it was found that the concomitant use of acid-suppressing drugs (PPIs and H_2_ receptor antagonists) as well as old age was a risk factor for enteropathy in NSAID users [27]. In such studies, we should consider confounding factors associated with PPI use that can lead to false-positive results; however, there are a number of prospective studies that report a high incidence of small intestinal damage in subjects receiving NSAIDs concomitantly with PPIs (Table 1) [16–18, 30]. Recently, a double-blinded, randomized trial showed that the incidence of small-bowel injury was 2.7 times higher in the celecoxib plus rabeprazole group than in the celecoxib plus placebo group [44]. However, it remains unclear if the aggravation of enteropathy by PPIs is clinically significant. Therefore, subsequent large-scale, double-blinded, randomized trials to identify the incidence of complicated intestinal ulcers are required.

Vonoprazan, which belongs to a class of acid-inhibitory agents called potassium-competitive acid blockers, was approved in Japan in February 2015, and its superiority or non-inferiority to PPIs for the treatment of acid-related diseases has been demonstrated [45–47]. Although vonoprazan suppresses gastric acid secretion by a different mechanism from PPIs, a recent animal study reported that both rabeprazole and vonoprazan aggravated NSAIDs-induced small intestinal injury in mice by reducing the population of *Lactobacillus johnsonii* in the small intestine [48]. Thus, strong inhibitors of gastric acid secretion may commonly cause small intestinal dysbiosis and the resultant aggravation of NSAIDs-induced enteropathy. However, to date, there are no clinical studies evaluating the effect of vonoprazan on the enteropathy.

Small intestinal bacterial overgrowth (SIBO) is characterized by a variety of clinical conditions associated with an excessive number of bacteria in the proximal small intestine. SIBO is associated with several conditions and diseases such as irritable bowel syndrome, which raise the question of the involvement of SIBO in NSAIDs-induced enteropathy. A cross-sectional study was conducted to evaluate the association between SIBO and the damage; the results revealed that SIBO, as diagnosed using a lactulose hydrogen breath test, was an independent risk factor for the development of severe small intestinal damage in chronic users of NSAIDs and LDA [49]. In addition to the effect on specific bacteria, PPIs may have the potential to induce SIBO [50]. PPIs may thus increase the risk of damage through dual mechanisms involving specific microbiome changes and SIBO.

Interestingly, the poor metabolizer genotype of CYP2C19 was a risk factor to the development of intestinal injury in a subject that was administered celecoxib plus rabeprazole. However, this was not observed in patients who were administered celecoxib alone [51]. Since this genotype causes poor metabolism of PPIs, strong inhibition of gastric acid secretion by PPIs in subjects carrying this genotype may lead to greater alteration of small intestinal flora, resulting in high sensitivity to the damage. In other studies, smoking, co-treatment of warfarin, and some single-nucleotide polymorphisms of CYP4F11 and CYP2D6 were considered as risk factors for LDA-induced small intestinal bleeding [52], whereas the presence of comorbidities (heart disease, chronic kidney disease, cirrhosis, chronic obstructive pulmonary disease, collagen disease, and malignant tumors) and the concomitant use of NSAIDs and LDA were associated with an increased risk for diaphragm disease [15]. However, most studies addressing risk factors involved a small number of subjects. Therefore, all risk factors referred to in this section need confirmation in a large-scale study.

## Pathophysiology of NSAIDs-induced enteropathy

### COX inhibition and topical effect

Similar to the upper GI tract, an important mechanism for the onset of NSAIDs-induced small intestinal damage is the inhibition of COXs. Since PGs also play a crucial role in the maintenance of intestinal integrity by upregulating mucosal blood flow and mucus/fluid and regulating intestinal motility [53–55], PG deficiency subsequent to COX inhibition leads to the impairment of the mucosal defensive system in the small bowel. In an animal study, neither SC-560 (a selective COX-1 inhibitor) nor rofecoxib (a selective COX-2 inhibitor) alone caused intestinal damage, but their combined administration induced lesions. Although PGs produced under healthy conditions are mostly derived from COX-1, these results suggest that COX-2-derived PGs also contribute to the defense system [56].

However, it is worth mentioning that unlike for the upper GI tract, PG deficiency alone is insufficient to cause small intestinal damage. The topical effect of NSAIDs, which is a COX-independent action that requires mucosal contact of the drug from the luminal side, is considered to play an important role as much as PG deficiency, in the early processes of injury [36, 57]. This direct action mostly involves the effects of NSAIDs absorbed into the epithelial cells on mitochondria. NSAIDs such as indomethacin, aspirin, naproxen, and piroxicam uncoupled the oxidative phosphorylation of isolated rat liver mitochondria and inhibited respiration in coupled mitochondria [58]. The oral administration of indomethacin induced mitochondrial morphological changes such as vacuolation, swelling, and loss of cristae, in the epithelial cells of the small intestine; these changes were also noted following the administration of dinitrophenol, a mitochondria uncoupling agent, indicating that these changes caused by indomethacin treatment are attributable to its activity to uncouple oxidative phosphorylation and/or inhibit electron transport. The mitochondrial morphological changes were reproduced by parenteral indomethacin, but they were absent in rats with a ligated bile duct [59]. Furthermore, aspirin exerted uncoupling activity in vitro, but oral aspirin, which is immediately absorbed in the upper GI tract without entering enterohepatic circulation, failed to induce morphological changes in mitochondria and small-bowel ulceration. On the contrary, when aspirin was administered directly into the small bowel, severe mucosal injury was induced in the area distal to the site of administration [58], which strongly suggests the indispensability of the topical effect during the induction of mitochondrial disorders. In a detailed analysis by Somasundaram et al. [60], both COX inhibition (PG deficiency) and mitochondrial disorders due to the uncoupling of oxidative phosphorylation have been shown to be required to induce small intestinal ulceration. They demonstrated that (1) dinitrophenol alone could elevate the permeability of the small bowel and induce mild neutrophil infiltration, but could not induce ulceration; (2) parenteral aspirin reduced the PG level in the small bowel, but it neither affected small-bowel permeability nor induced ulceration and inflammation; and (3) treatment with dinitrophenol in combination with parenteral aspirin resulted in ulceration with increases in permeability and mucosal inflammation and decrease in PG levels, whose phenomena are similar to those observed in oral indomethacin.

A growing body of evidence is accumulating to demonstrate that the uncoupling activity of NSAIDs is mainly attributed to the opening of the mega-channel called mitochondrial permeability transition pore (PTP), which is composed of proteins that link the inner and outer mitochondrial membranes [61–63]. The opening of PTP is linked to mitochondrial dysfunction associated with mitochondrial depolarization, cessation of ATP synthesis, Ca^2 +^ release, and inhibition of respiration [64]. This opening also allows low-molecular-weight substrates less than 1500 Da to freely penetrate the mitochondrial matrix, leading to mitochondrial swelling and cell deaths (apoptosis or necrosis) through the release of cytochrome c into the cytosol [36]. In isolated rat mitochondria, diclofenac induced mitochondrial swelling, depolarization of membranes, Ca^2+^ leakage, and oxidation of nicotinamide adenine dinucleotide phosphate and protein thiol. All these phenomena were suppressed by the coincubation of the mitochondria with cyclosporin A, an inhibitor of PTP [61]. In addition, the chemical inhibition or genetic deletion of mitochondrial cyclophilin D, a critical regulator of the PTP, prevented diclofenac-induced small intestinal ulceration in mice [65], confirming significant contribution of PTP in NSAIDs-induced enteropathy.

As a result of such initial disorders brought by COX inhibition and the topical effects on mitochondria, intestinal permeability is increased with the disruption of the barrier function, which facilitates the invasion of the small bowel by luminal injury factors such as enterobacteria and bile. Furthermore, recent studies have suggested that dietary factors are involved in increased intestinal permeability during the development of NSAIDs-induced damage: Insoluble dietary fibers contribute to the surface damage of the intestinal epithelium when the mucus is decreased by NSAIDs’ administration [66], and gliadin, a component of wheat gluten, increases the intestinal permeability via the epidermal growth factor receptor-dependent signaling pathway [67]. All these events lead to promoting a series of inflammatory events sufficient for inducing macroscopic ulceration.

### Enterobacteria and bile

Enteric bacteria play a crucial role in NSAIDs-induced small intestinal ulceration. Germ‐free rats treated with indomethacin did not develop intestinal ulcers. However, when *Escherichia coli* were reintroduced to these rats, they became susceptible to this intestinal damage [68]. Ampicillin, a broad-spectrum antibiotic, markedly inhibited indomethacin-induced enteropathy with a decrease in the number of enterobacteria invading the intestinal mucosa [69]. Aztreonam (specific for Gram-negative bacteria) protected indomethacin-induced damage to a similar extent as ampicillin), whereas vancomycin (specific for Gram-positive bacteria) had no effect [70]. In addition, NSAIDs caused an increase in Gram-negative bacterial numbers in the small intestine during the development of the injury [71, 72] and psychological stress, which led to elevated proportion of Gram-negative bacteria, γ-*Proteobacteria* and *Bacteroidetes*, aggravated indomethacin-induced damage [73]. Although recent microbiome analyses revealed that NSAIDs induce various types of dysbiosis in the small intestine, including an increase in some Gram-positive bacteria such as *Clostridium* spp [74] and *Enterococci *[75], it is strongly conceivable that among all bacteria, Gram-negative enteric bacteria play a major role in the development of small intestinal ulcers.

Bile appears to have an important role in the pathogenesis of small-bowel damage, because NSAIDs did not induce macroscopic intestinal injury, despite the induction of permeability and inflammation in bile duct–ligated rats [59]. Pathogenic roles of bile acids for the damage have been demonstrated in both in vivo and in vitro studies. Ursodeoxycholic acid increased intestinal inflammation caused by indomethacin in rats [76], although another study demonstrated the opposite effect [77]. In an in vitro study using gastric AGS and intestinal IEC-6 cells, combinations of bile acid (deoxycholic acid, taurodeoxycholic acid, or glycodeoxycholic acid) and indomethacin increased cell plasma membrane permeability and became more cytotoxic than these agents alone [78]. Although the precise mechanisms of the damage by bile acid are still unclear, some bile acids including deoxycholic acid and taurodeoxycholate have been shown to induce a pro-inflammatory cytokine, interleukin (IL)-8, and activate nuclear factor-κB (NF-κB) in HT29 and IEC-6 cells [79, 80]. In addition, bile acids such as chenodeoxycholate are known to open PTP [81]. However, considering that in bile duct-ligated rats, the administration of chenodeoxycholic acid along with the indomethacin failed to produce macroscopic ulceration, there may exist another component of bile secretion that is important for the induction of the injury.

The finding that the ulcerogenic effect of NSAIDs on the small bowel is abolished by bile duct-ligation suggests two possible mechanisms for this bile-mediated damage. First, bile components including bile acids are luminal aggressive factors for the pathogenesis of NSAIDs-induced enteropathy, as described above. Second, the enterohepatic circulation of NSAIDs plays a crucial role in the pathogenesis of the damage. In other words, the protection of the damage by bile duct ligation results from the elimination of the chance that NSAIDs exert the topical effect. NSAIDs that do not undergo enterohepatic circulation do not cause significant intestinal ulceration [58, 71]. Many NSAIDs are carboxylic acids that are conjugated in the liver to acyl glucuronides. The acyl glucuronides of NSAIDs are excreted across the hepatocanalicular membrane into bile. Then, these conjugates are enzymatically cleaved by bacterial β-glucuronidases in the lumen of the small bowel and aglycones are reabsorbed. This enterohepatic circulation results in repeated and prolonged exposure of the gut mucosa to NSAIDs, which provides sufficient topical effect on the epithelial cells for the induction of intestinal damage. The pathological importance of the enterohepatic circulation and bacterial β-glucuronidases is further supported by findings that hepatocanalicular conjugate export pump-deficient rats, that cannot export glucuronide NSAIDs into bile and deliver these glucuronides to the gut lumen, exhibited markedly less severe intestinal damage caused by diclofenac [82] and pharmacologic targeting of luminal bacterial β-glucuronidase by a specific inhibitor protected against diclofenac-induced enteropathy [65]. While, as described below, enteric bacteria are a key player for induction of inflammatory responses in NSAIDs-induced enteropathy, the latter finding indicates that gut microbiota contribute twofold in the pathogenesis of the damage, namely the involvement of the enterohepatic circulation of NSAIDs and the activation of innate immune systems.

### Activation of innate immunity by Gram-negative bacteria and other factors

Bertrand et al. reported that indomethacin treatment induced the overexpression of tumor necrosis factor-α (TNF-α) in the small intestine, which was associated with the onset of the intestinal macroscopic ulcerations, and preceded an increase in myeloperoxidase (MPO) activity, a marker for neutrophil infiltration [83]. The inhibitors of TNF-α and of IL-1β/TNF-α prevented intestinal damage induced by indomethacin, with the suppression of the increase in MPO activity [69, 83]. Together with the findings that ampicillin inhibited indomethacin-induced small intestinal damage, and this was associated with decreases in the number of enterobacteria invading the intestinal mucosa and MPO activity [69], these results strongly suggested the importance of enteric bacteria in triggering the inflammatory cascades during the development of NSAIDs-induced enteropathy.

The Toll-like receptor (TLR) family plays a crucial role in innate immune responses against microbial pathogens, as well as in the subsequent induction of adaptive immune responses. TLRs recognize the specific molecular patterns found in a broad range of microbial pathogens, known as pathogen-associated molecular patterns (PAMPs). To date, 10 and 12 functional TLRs have been identified in humans and mice, respectively [84]. Each TLR detects distinct PAMPs derived from viruses, bacteria, mycobacteria, fungi, and parasites. For example, TLR4 was found to be a receptor for lipopolysaccharide (LPS), a major cell wall component of Gram-negative bacteria [85], and to require MD-2 to respond efficiently to LPS [86]. TLR2 in combination with TLR1 recognizes lipoteichoic acid, a major constituent of the cell wall of Gram-positive bacteria [86]. Ligand binding to TLRs activates downstream signaling pathways, including NF-κB, mitogen-activated protein kinases, and type I interferon pathways, which induces pro-inflammatory cytokines and chemokines and eradicates invading pathogens.

Consistent with the above-mentioned results indicating that antibiotics that are active for Gram-negative bacteria protected against NSAIDs-induced small damages, the damage induced by indomethacin or diclofenac was markedly inhibited in TLR4-mutant mice, being accompanied with decreases in inflammatory cytokines expressions including those of TNF-α, monocyte chemoattractant protein-1, and keratinocyte chemoattractant [70]. LPS 1 h after indomethacin aggravated indomethacin-induced damage, whereas pretreatment with LPS inhibited the damage with the reduction of the TLR4 expression [70]. This phenomenon seems to result from the development of endotoxin tolerance, where prior exposure to LPS induces a transient state of cell refractoriness to subsequent LPS exposure [87]. Interestingly, pretreatment with TLR2 agonists also attenuates indomethacin-induced small intestinal lesions by suppressing the TLR4 signaling pathway [88], which is attributed to the occurrence of cross-tolerance between TLR2 and TLR4 ligands [89]. Although two signaling pathways, the MyD88-dependent and MyD88-independent pathways, have been described following TLR4 activation [90], MyD88^−/−^ mice exhibited resistance to NSAIDs-induced damage at a similar level to TLR4^−/−^ mice. Therefore, the TLR4/MyD88 axis plays a key role in the development of NSAIDs-induced enteropathy.

In addition to the recognition of PAMPs, TLR2, TLR4, and TLR9 have also been shown to recognize endogenous ligands, which have been termed danger-associated molecular patterns (DAMPs). High mobility group box 1 (HMGB1), a DAMP which leaks out of cells during necrotic cell death, and is actively secreted by monocytes, exerts pro-inflammatory actions via TLR2 and the receptor for advanced glycation end-products (RAGE) as well as TLR4 [91]. In NSAIDs-induced enteropathy, the prominent cytoplasmic staining of HMGB1 in damaged epithelial cells was observed and recombinant HMGB1 aggravated the damage through the activation of NF-κB and mitogen-activated protein kinases [92]. In addition to TLR4, TLR2 and RAGE are expressed in the small intestine [92], and TLR2 as well as TLR4 was up-regulated both in the ileum and the ceco-colonic region after indomethacin administration [74]. However, neither TLR2 deficiency nor RAGE deficiency affected the severity of the NSAIDs-induced damage. Furthermore, exogenous HMGB1 also aggravated NSAIDs-induced small intestinal damage in both TLR2^−/−^ and RAGE^−/−^ mice and increased the mRNA expression levels of TNF-α in these mice, but failed to affect the damage and mRNA expression levels of TNF-α in TLR4^−/−^ mice, suggesting that HMGB1 released from the damaged epithelial cells promoted NSAIDs-induced damage through TLR4. Thus, both exogenous and endogenous TLR4 ligands act in concert to elicit the intestinal inflammation that drives the enteropathy.

**Fig. 2:**
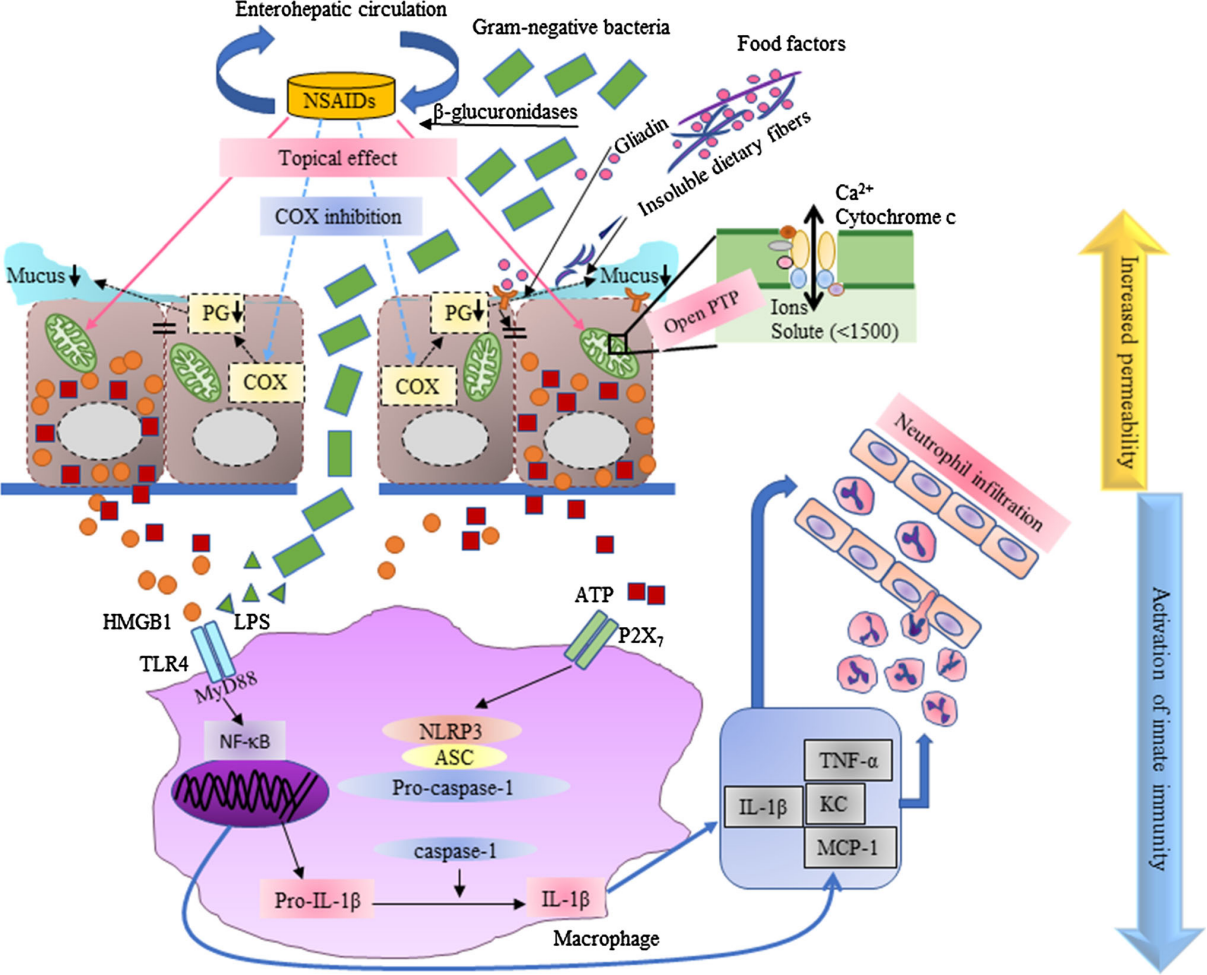
The mechanism of increased intestinal permeability and activation of innate immune system during development of NSAIDs-induced small intestinal damage. *NSAID* Non-steroidal anti-inflammatory drug, *COX* cyclooxygenase, *PG* prostaglandin, *PTP* permeability transition pore, *HMGB1* high mobility group box 1; LPS, lipopolysaccharide, *TLR4* Toll-like receptor 4, *NF-κB* nuclear factor-κB, *NLRP3* NLR family pyrin domain containing 3 protein, *IL-1* interleukin-1, *TNF-α* tumor necrosis factor-α, *KC* keratinocyte chemoattractant, *MCP-1* monocyte chemoattractant protein-1

A recent study demonstrated that the inflammatory signals triggered by NSAIDs also activated the NLR family pyrin domain containing 3 protein (NLRP3) inflammasome [93], which comprises NLRP3, apoptosis-associated speck-like protein containing a caspase recruitment domain (an adaptor protein), and pro-caspase-1. Recognition of endogenous and exogenous signals arising from intracellular or extracellular stressors by NLRP3 triggers the assembly of the inflammasome, leading to the cleavage and activation of pro-caspase-1 [94]. Once caspase-1 is activated, it promotes the processing of pro-IL-1β and pro-IL-18 into their mature active forms. Treatment with recombinant IL-1β aggravated NSAIDs-induced intestinal damage, while the in vivo blocking of IL-1β using neutralizing antibodies inhibited it. Furthermore, NLRP3^−/−^ and caspase-1^−/−^ mice exhibited less severe damage and lower production levels of mature IL-1β [93], suggesting that the NLRP3-derived IL-1β as well as TNF-α and monocyte chemoattractant protein 1 mediates the inflammatory cascades and the damage. Both TLR4-dependent signaling and P2X_7_-dependent signaling (which is stimulated by extracellular ATP from damaged epithelial cells) are required for NLPR3 activation [93]. Given that both TLR4 and NLRP3 inflammasome are mainly expressed on macrophages in the small intestine during development of the damage [70, 92, 93], the macrophage-mediated activation of innate immune systems and the resultant neutrophil infiltration are key events in the late phase of NSAIDs-induced intestinal ulceration (Fig. 2).

## Prophylaxis and treatment

Since as described above, there exist several steps for the complete development of NSAIDs-induced intestinal ulceration, drugs which interfere with one of these steps could be useful for preventing and treating NSAIDs-induced enteropathy. The efficacy of some drugs including misoprostol, metronidazole, and sulphasalazine, had been reported more than 2 decades ago [95–97]. However, in these studies, efficacy was indirectly evaluated by measuring several markers such as hemoglobin levels and fecal excretion of radio-labeled neutrophils. Currently, we can directly evaluate the effect of drugs on the enteropathy by small intestinal endoscopy, and the utility of several drugs have been demonstrated in clinical trials using capsule endoscopy. Although the recommended treatment for patients with NSAIDs/LDA-induced enteropathy is the withdrawal of these drugs, cessation of NSAIDs often results in recurrence of severe pain. Furthermore, several reports indicated that in LDA users with cardiovascular diseases who developed GI bleeding, discontinuation of LDA was associated with poor prognosis such as high mortality rate [98, 99]. Therefore, many patients with such enteropathy cannot discontinue NSAIDs or LDA. Prophylactic drugs and drugs that exert the healing effect under the continuation of NSAIDs or LDA are essential.

### Misoprostol

Since PG deficiency is the key mechanism by which NSAIDs induce enteropathy, PG supplementation is thought to be the most reasonable therapy. Misoprostol, a synthetic PGE_1_ analogue, is the first drug whose healing effect on LDA-induced small intestinal damage had been demonstrated in a clinical study using capsule endoscopy [37]. Furthermore, this drug also exerted a prophylactic effect against small intestinal lesions caused by a 2-week administration of diclofenac [23]. Recently, a randomized trial reported that misoprostol was effective in healing small-bowel ulcers [100]. However, this study recruited both NSAIDs and aspirin users with occult bleeding only. Furthermore, it is unclear whether patients in this study continued aspirin or NSAIDs during the ulcer healing period. More recently, Kyaw et al. reported that misoprostol was superior to the placebo in the promoting healing of small-bowel ulcers and improving anemia among LDA users complicated by small-bowel ulcer bleeding with a complete ulcer healing rate of the misoprostol group and placebo group being 28.6% and 9.5%, respectively [101]. This is the first randomized study on the treatment of small-bowel bleeding while continuing LDA. Although it needs to be determined if this result can be generalized to other NSAIDs except for LDA, misoprostol should be used as a first choice for treating NSAIDs/LDA-induced enteropathy.

### Antibiotics and probiotics

Scarpignato et al. conducted a placebo-controlled study using capsule endoscopy to determine whether rifaximin, a poorly absorbed antibiotic, has a prophylactic effect against intestinal lesions induced by a 2-week administration of diclofenac in healthy volunteers, and they reported that rifaximin reduced the mean number of lesions and prevented the development of the larger lesions or ulcers, although they could not find significant difference in the proportion of subjects who developed at least 1 mucosal break, which was a primary endpoint [102]. A prospective, large trial including patients in long-term NSAIDs therapy is required to evaluate the efficacy of antibiotics in real clinical settings.

The use of probiotics is another promising therapy to treat NSAIDs-induced enteropathy by modulating the bacteria-triggered pathogenic processes. Capsule endoscopic studies showed a significant healing effect of *L. casei* and *L. gasseri* against the enteropathy in chronic LDA users [103, 104]. VSL#3, a probiotic mixture, reduced the indomethacin-induced increase in fecal calprotectin concentrations in healthy subjects, but the efficacy was not evaluated endoscopically [105]. The precise mechanisms of the beneficial effects of these probiotics remain unclear, but besides the direct effect on intestinal bacteria, the anti-inflammatory properties of bacterial metabolites seem to contribute to the efficacy against NSAIDs-induced small intestinal damage. For example, lactic acid produced by *L. casei* strain Shirota prevented the LPS-triggered activation of NF-κB and mitogen-activated protein kinases in macrophages [106]. Since probiotics are generally safe and well tolerated, a large-scale, high-quality trial to evaluate the effect of probiotics on the NSAIDs/LDA-induced intestinal damage is warranted.

### Anti-cytokine therapy

In rats, the *in vivo* blocking of TNF-α by neutralizing antibodies provided a significant reduction in indomethacin-induced small intestinal damage [70], and TNF-α knockout mice exhibited less severe indomethacin-induced damage, with a reduction in neutrophil infiltration and epithelial cell apoptosis [107]. A clinical study using a propensity matching method demonstrated the marked reduction of the risk for NSAIDs-induced severe enteropathy in RA patients with anti-TNF therapy [108]. Thus, anti-TNF biological agents are a candidate for treating the enteropathy, but the high cost of using these agents limits clinical trials and further research studies. As described above, the NLRP3 inflammasome/IL-1β axis could be a target for the treatment of NSAIDs-induced enteropathy. In mice, colchicine prevented NSAIDs-induced small intestinal damage by suppressing the activation of the NLRP3 inflammasome and subsequent mature Il-1β production [109]. As colchicine production is inexpensive, and it has been used for treating many patients with gout and other diseases, a clinical trial should be urgently performed to prove the efficacy of colchicine for treating enteropathy.

### Gastric muco-protective drugs

Rebamipide, a muco-protective drug, has been clinically proven to effectively heal gastric ulcers and prevent NSAIDs-induced gastroduodenal damage [110]. This drug possesses various effects on the GI tract, including the induction of COX-2 [111], inhibition of inflammatory cytokine expression [112] and NSAIDs-induced PTP [113], and the modulation of the gut microbiome [114]. Studies using capsule endoscopy have shown that rebamipide also prevented both NSAIDs- and LDA-induced enteropathy in healthy volunteers [22, 115] and promoted the healing of intestinal mucosal breaks in chronic users of NSAIDs and LDA [116, 117]. Other muco-protective drugs such as irsogladine [118], polaprezinc [119], geranylgeranylacetone [120], and ecabet sodium [121] have demonstrated their therapeutic potential for NSAIDs/LDA-induced enteropathy. However, similar to other drugs besides misoprostol, none of these muco-protective drugs have been proven to be effective on clinically significant small intestinal injuries (Table 2).

**Table 2:** Drugs whose effects on NSAIDs/LDA-induced enteropathy have been demonstrated in capsule endoscopic studies

Drug	Primary prophylactic effect		Healing effect			
			Non-bleeding lesions		Bleeding ulcers	
	NSAID	LDA	NSAID	LDA	NSAID	LDA
Misoprostol	◎ [23]		◎ [100]	◎ [100] ○ [37]		◎ [101]
Antibiotics						
Rifaximin	◎ [102]					
Probiotics						
*Lactobacillus casei*				◎ [103]		
*Lactobacillus gasseri*				◎ [104]		
*Bifidobacterium breve*		◎ [122]				
Anti-TNF-α	○ [108]					
Muco-protective drugs						
Rebamipide	◎[22]	◎[115]	◎ [117]	◎[116, 117]		
Irsogladine	◎ [118]					
Polaprezinc				◎ [119]		
Geranylgeranylacetone	◎ [120]					
Ecabet sodium		◎ [121]				

◎ Randomized controlled trial. ○ Non-randomized controlled trial. The reference numbers are shown in brackets

*NSAID* non-steroidal anti-inflammatory drug, *LDA* low-dose aspirin, *TNF* tumor necrosis factor

## Future perspectives

The role of gut microbiota in NSAID-induced small-bowel damage remains a largely unexplored area. In a small-scale randomized trial, Endo et al. reported that *L. casei* reduced small-bowel endoscopic injury among chronic NSAID users [103]. Recently, a double-blind randomized trial of healthy volunteers showed a significant reduction in LDA-induced small-bowel mucosal injury with oral *Bifidobacterium breve* (Bif195) [122]. To identify whether there are specific patterns of microbial profile among chronic NSAID users, we need to use bioinformatics to identify subpopulations that are associated with certain phenotypes (e.g., bleeding, stricture, and protein-losing enteropathy). With better understanding of our gut microbiota, we may be able to identify certain microbial footprints that predispose to small-bowel enteropathy in the future.

## Abbreviations

NSAID: Non-steroidal anti-inflammatory drug
RA: Rheumatoid arthritis
COX: Cyclooxygenase
PG: Prostaglandin
GI: Gastrointestinal
PPI: Proton pump inhibitor
SIBO: Small intestinal bacterial overgrowth
PTP: Permeability transition pore
IL: Interleukin
NF-κB: Nuclear factor-κB
TNF-α: Tumor necrosis factor-α
MPO: Myeloperoxidase
TLR: Toll-like receptor
PAMP: Pathogen-associated molecular patterns
LPS: Lipopolysaccharide
HMGB1: High mobility group box 1
DAMP: Danger-associated molecular patterns
RAGE: Receptor for advanced glycation end-products
NLRP3: NLR family pyrin domain containing 3 protein
